# Dermatophyte resistance – on the rise

**DOI:** 10.1186/s13047-023-00665-5

**Published:** 2023-10-05

**Authors:** Ivan R. Bristow, Lovleen Tina Joshi

**Affiliations:** 1 AECC University College, Parkwood Campus, Parkwood Road, Bournemouth, BH5 2DF UK; 2 Molecular Microbiology, Peninsula Dental School, University of Plymouth, Drake Circus, Plymouth, PL4 8AA UK

**Keywords:** Dermatophyte, Infection, Drug resistance, Terbinafine

## Abstract

Dermatophytes are group of filamentous fungi which have adapted to living on the skin of humans and other animals. In the last decade, reports have emerged from Asia of new dermatophyte strains showing resistance to the commonly used antifungal agent terbinafine and others. The spread of these resistant strains has been noted in many other countries globally. Little is known about the mechanisms or management of this emerging problem. Urgent research and changes to current practice are required if the spread of the infection is to be contained and managed effectively.

## Background

The issue of antibiotic resistance in the fight against infectious diseases has long been recognised and highlighted in the literature. However, whilst much attention has focussed on bacterial agents, only recently has awareness been raised internationally about the growing threat of fungal resistance to common antifungal agents. Dermatophytes – the causative agents for common skin, hair and nail infections have been regarded as treatable, minor infections causing discomfort and irritation but to the immunosuppressed they can represent a serious, invasive threat to human health. Around 25% of the world’s population is estimated to be affected by superficial dermatophyte infection at some point in their lives [1]. In the last decade, reports began to emerge, initially from India, describing a change in the pattern of dermatophytosis with *Trichophyton mentagrophytes* & *T*. *interdigitale* becoming the dominant strains over *T. rubrum* [2], and showing increased resistance to standard treatment regimes.

## Main body

A paper published in 2018 reported the increase in stubborn superficial dermatophyte infections occurring in India, caused by a variant identified as *T. mentagrophytes* type VIII [3]. Due to distinct genetic differences, it was renamed as *T. indotineae*, a novel species showing high terbinafine resistance [4, 5] and being easily transmissible from human to human. A microbiological study from across the same country examined samples from 402 patients identifying an alarmingly high rate (78%) of *T. indotineae* with 71% of isolates demonstrating terbinafine resistance [6].

Analysis of terbinafine resistant strains demonstrate several point mutations in the gene encoding for squalene epoxidase [7] – an enzyme responsible in fungal cell wall formation and the target for terbinafine antifungal therapy. Inhibition by antifungal agents leads to an accumulation of squalene and a depletion in the levels of ergosterol, leading to a lack of fungal cell growth [8]. The reasons for the emergence of resistant strains have not been fully researched, but in India, potent steroid creams combined with antifungal agents and antimicrobial agents are freely available and have been sold in large quantities over the counter [9]. These preparations actually being cheaper than pure antifungal agents mean they are the often first choice for consumers [10]. Topical steroids suppress the normal immune response and act quickly to improve a patient’s symptoms but do little to eradicate the infection even when combined with an antifungal due to the steroid’s high potency. Without education and regulation, patients may also be applying these potent steroids for months or even years [2] with an underlying fungal skin infection. Consequently, fungal skin infection rates are high, have an unusual clinical presentation (making diagnosis more difficult), and are conducive to developing resistance [2].

Published reports have demonstrated the spread of the species across the world from within Asia [11] to North America and Europe [12–14] probably facilitated through global travel. A 2019 survey of 39 mycologists in 23 European countries was undertaken to explore the extent of antifungal resistance in dermatophytes – 85% (17/20 countries) had observed or mycologically confirmed antifungal resistance (a total of 126 cases) affecting the scalp, body, groin and plantar surfaces [15]. This year, Gupta et al. [16] analysed 5432 toenail clippings from patients across the US with suspected onychomycosis and demonstrated a terbinafine conferring mutation rate of 3.7%.

The picture that is currently emerging is a cause for concern with a rapid spread of terbinafine resistant dermatophytes across continents. Unlike bacterial infections, many superficial fungal infections are treated empirically in the clinic without formal laboratory identification. Consequently, the true extent of the problem may be masked. Moreover, emerging resistant strains such as *T. indotineae* may be clinically indistinguishable from strains of *T. mentagrophytes* and *interdigitale* [5]. Specialist molecular genetic testing, such as Polymerase Chain Reaction (PCR), would be required to identify such strains of the dermatophyte along with phenotypic antimicrobial susceptibility testing to a range of common antifungal agents to ascertain the Minimum Inhibitory Concentration (MIC) required to inhibit fungal growth and thus properly inform practice.

Urgent research is required at this stage to fully understand how antifungal resistance has arisen along with investigations into which other antifungal agents may be used safely and effectively. For example, azoles such as clotrimazole and miconazole and newer agents in the azole class (luliconazole) or the development of effective antimicrobial combination therapies. Clinicians should remain vigilant to the problem and when extensive cases of dermatophyte disease arise on the foot or in the toenails, which fails to respond to antifungal therapy, consider this a possibility (along with reinfection and relapse) and where required refer patients to mycology for antifungal susceptibility testing in combination with specialist molecular testing to identify the causative agents where available. However, not all laboratories perform routine antifungal testing with MICs, coupled with molecular testing, primarily because of the incubation complexities involved in aseptic fungal culture. This means that many cases remain undetected or misdiagnosed, which could contribute to potentiation of further resistance.

Surveillance of these cases is important in understanding the scale of antifungal resistance in dermatophytes and to update diagnostic process. Given that mycological culture is not routinely performed within laboratories, clinicians who suspect terbinafine resistance should seek to prescribe combinations of antifungal medications such to treat infection and limit resistance to a single antimicrobial agent. This antimicrobial stewardship will help to reduce spread of antimicrobial resistant dermatophytes. Clinicians can also encourage patients to adopt infection prevention and control measures to limit the spread of infection. This includes regularly attending podiatrist appointments to monitor foot health, using antiseptic washes to treat infection (such as hypochlorous acid), washing socks regularly with disinfectant.

## Conclusion

There is an urgent need to understand the burden of terbinafine resistance within dermatophyte strains. Clinical diagnosis should consider terbinafine resistance as a possible cause of failure to respond to antifungal therapy and use molecular and phenotypic diagnostic methods to identify the causative agent of infection; thus optimising effective antifungal use in patients.

## Data Availability

Not applicable.
